# Haploid induction: an overview of parental factor manipulation during seed formation

**DOI:** 10.3389/fpls.2024.1439350

**Published:** 2024-09-04

**Authors:** Jingpu Song, Raju Datla, Jitao Zou, Daoquan Xiang

**Affiliations:** ^1^ Aquatic and Crop Resource Development Research Centre, National Research Council of Canada, Saskatoon, SK, Canada; ^2^ Global Institute for Food Security, University of Saskatchewan, Saskatoon, SK, Canada

**Keywords:** haploid induction, plant breeding, seed development, maternal factors, paternal factors

## Abstract

In plants, *in vivo* haploid induction has gained increasing attention for its significant potential applications in crop breeding and genetic research. This strategy reduces the chromosome number in progeny after fertilization, enabling the rapid production of homozygous plants through double haploidization, contrasting with traditional inbreeding over successive generations. Haploidy typically initiates at the onset of seed development, with several key genes identified as paternal or maternal factors that play critical roles during meiosis, fertilization, gamete communication, and chromosome integrity maintenance. The insights gained have led to the development of efficient haploid inducer lines. However, the molecular and genetic mechanisms underlying these factors vary considerably, making it challenging to create broadly applicable haploidy induction systems for plants. In this minireview, we summarize recent discoveries and advances in paternal and maternal haploid induction factors, examining their current understanding and functionalities to further develop efficient haploid inducer systems through the application of parental factor manipulation.

## 1 Introduction

Haploid plants inherit a single set of chromosomes from either male (sperm cells) or female (egg cells) gametes, classified as maternal or paternal haploids depending on their genome origin, respectively. The chromosome set of a haploid plant can be spontaneously doubled or artificially doubled through chemical treatments, resulting in the production of double haploid (DH) lines that are completely homozygous at all loci via the chromosome doubling process. Compared to broadly applied conventional breeding approaches such as backcrossing or selfing, the production of double haploid (DH) plants through haploid induction stands out as one of the most efficient breeding strategies to develop genetically stable and breeding-ready lines in modern agriculture (Chen et al., 2023; Flores-Tornero et al., 2023). Furthermore, the use of *in vivo* haploid technology empowers plant breeders to significantly reduce the time and cost associated with cultivar development compared to conventional breeding processes. Consequently, recent advancements in this technology are garnering significant attention in applied plant biology research.

Haploid plants are primarily produced through two distinct approaches: *in vitro* and *in vivo* methods (Seguí-Simarro et al., 2021). *In vitro* methods involve culturing haploid gametophytic cells, which then differentiate into haploid embryos and eventually develop into haploid plants. This technique utilizes either microspores (pollen) or megaspores (ovules), depending on the cell responsiveness within a given species. However, *In vitro* methods face significant challenges due to their strong dependence on genotype of plant species and the high risk of mutations occurring during cultivation (Seguí-Simarro et al., 2021). Additional obstacles include the requirement for specialized expertise in precise anther staging, anther pre-treatment procedures, and culture media formulation. On the other hand, *in vivo* methods utilize specific pollination techniques, including irradiated pollen, inter-specific crosses, and crosses with inducer lines during the reproductive phase of plant development. *In vivo* haploid induction shows promise in circumventing limitations such as low efficiency and longer processing times associated with *in vitro* culture systems, which are applicable to only a limited number of species (Flores-Tornero et al., 2023). *In vivo* methods employ haploid inducer (HI) lines that may also involve double fertilization event, unique sexual reproduction process in flowering plants. During double fertilization, one sperm cell fuses with an egg cell, forming a diploid embryo, while the second sperm cell combines with a binucleate central cell, resulting in a triploid endosperm. Fertilization with an HI plant can yield a haploid embryo either through single fertilization (sperm cell fusion with the central cell) or through post-zygote genome elimination in double fertilization, contingent upon the specific HI lines utilized (Sarkar and Coe, 1966; Zhao et al., 2013; Ishii et al., 2016; Gilles et al., 2017; Chen et al., 2023). In this mini-review, we have summarized key recent advances highlighting the understanding of maternal and paternal genetic factors that exhibit diverse haploid induction efficiencies. Moreover, exploiting the emerging potential of both paternal and maternal haploid-inducing factors presents new opportunities to gain deeper insights into their functions for broadening their applications. This strategic exploration will offer promise for advancing the development of efficient haploid seed production systems and their utilization in plant breeding programs.

## 2 Paternal factors for haploid induction

The maize Stock6, identified as the first paternal HI line in 1959, has been widely recognized as a highly effective system for DH (doubled haploid) production in maize breeding programs (Coe, 1959; Liu et al., 2017). Despite its longstanding utility, the genetic and biological mechanisms underlying the HI trait in this line remained elusive until recent breakthroughs. Three research groups elucidated that the HI phenotype of Stock6 is caused by a 4-bp insertion within *ZmPLA1/MTL*, the gene encoding a phospholipase A exclusively localized to sperm cytoplasm (Gilles et al., 2017; Kelliher et al., 2017; Liu et al., 2017). Subsequent studies, however, demonstrated that *NLD* does not localize to the cytosol and plasma membrane of sperm cells, but rather to the pollen endo-plasma membrane (endo-PM) (Gilles et al., 2021). The underlying molecular mechanism was further elucidated through the application of multiple omics techniques using zmpla1 mutant anthers. The analysis of comprehensive datasets suggested a critical role for the burst of reactive oxygen species (ROS) in haploid induction (HI) (Jiang et al., 2022). In this study, maize pollen was treated with methimazole or phosphatidylcholine, leading to the transfer of sperm with ROS-fragmented genomes into the embryo sac via the elongation of the pollen tube. This process stimulates the development of female gametes to produce haploid embryos, providing a simple HI method that does not require inducer lines and could potentially be expanded to other species (Jiang et al., 2022). Jiang et al. proposed that the absence of functional ZmPLA1 in pollen leads to elevated levels of phosphatidylcholine in sperm cells, which triggers reactive oxygen species (ROS) production, resulting in sperm DNA breakage and fragmentation post-double fertilization. Consequently, the loss of the paternal genome in the zygote culminates in the generation of haploid seeds. Additionally, this study has unveiled ZmPOD65, a sperm-specific peroxidase, as a novel paternal factor crucial for haploid induction (HI) in maize. The discovery of this new gene controlling HI and the development of a novel HI method for maize underscore the significance of maintaining a balanced level of reactive oxygen species (ROS) to ensure normal reproduction. Comparative analyses of wild-type and CRISPR/Cas9-edited matrilineal mutant (*mMTL*) wheat pollens revealed significant differences. The *mMTL* pollen exhibited a second reactive oxygen species (ROS) wave at the three-nuclei stage, accompanied by decreased antioxidant enzyme activity. RNA sequencing uncovered altered expression patterns in genes associated with fatty acid biosynthesis and ROS homeostasis and proved that the *mtl* mutation resulted in ROS accumulation and DNA fragmentation during late pollen development (Sun et al., 2022). These findings will likely open new additional promising avenues to expedite HI based crop breeding efforts.

*Maize PHOSPHOLIPASE D3* (*ZmPLD3*) encodes another phospholipase belonging to the phospholipase D subfamily. Mutations in *ZmPLD3* trigger maternal HI in maize, demonstrating synergistic effects rather than functional redundancy with *zmpla1* (Li et al., 2021). Both *ZmPLD3* and *ZmPLA1* are specifically expressed in the pollen, and the loss of either gene’s function triggers maternal HI. However, it remains unclear whether the haploid seed resulting from zmpld3-induced HI stems from a single or double fertilization event.

*ZmPLA1* is highly conserved in monocots and the application of HI technology with *ZmPLA1* has been extended to other monocots, for example, rice and wheat (Yao et al., 2018; Liu et al., 2020). Intriguingly, no functional orthologs have been identified in dicots. However, another maize HI-trigger gene *ZmDMP* (*DOMAIN OF UNKNOWN FUNCTION 679* membrane protein) is widely present in both monocots and dicots (Zhong et al., 2019, 2020; Wang W. et al., 2022). In *Arabidopsis*, loss-of-function mutations in *DMP8* and *DMP9*, two *ZmDMP*-like genes, result in haploids (Takahashi et al., 2018; Cyprys et al., 2019; Zhong et al., 2020). The two sperm membrane proteins DMP8 and DMP9, play a crucial role in egg cell-derived signals that facilitate the translocation of *GCS1* (*GENERATIVE CELL SPECIFIC 1*) from internal storage vesicles to the sperm plasma membrane, ensuring successful sperm and egg cell fusion (Wang W. et al., 2022). Knocking down the *DMP9* gene in *Arabidopsis* leads to approximately 17% single fertilization of the central cell, while the double mutant *dmp8/9* elevates the single central cell fertilization rate of the *dmp9* single mutant by about 2% (Cyprys et al., 2019; Wang W. et al., 2022). Research by Wang N. et al. (2022) showed that simultaneously inactivating the *MtDMP8* and *MtDMP9* genes can trigger *in vivo* maternal haploid induction in this species. This successful haploid induction in *M. truncatula* opens up promising avenues for legume haploid gene editing and for studying the mechanisms of haploid induction in legumes. Maternal haploids can be produced through the single fertilization of the central cell. The applicability of this haploid induction strategy has been further demonstrated in other plant species, as evidenced by Chen et al. (2023), who successfully used it to produce haploids in watermelon. Research into *DMP* genes’ function during double fertilization has laid a crucial foundation for comprehending how these genes trigger haploid embryo development. To enhance haploid induction efficiency, future work should focus on creating *dmp* mutants across various plant species and implementing HI enhancer screens to identify modifier genes. Additionally, the potential of *dmp* system lines for producing gene-edited maternal haploids, especially in transformation-resistant genotypes, remains an important area for investigation.

## 3 Maternal factors for induction of haploids

Recent studies have shown that several maternal factors with loss of function mutations result in production of haploid seeds (**Table 1**) (Flores-Tornero et al., 2023; Jang et al., 2023; Mao et al., 2023; Zhang et al., 2023). In *Arabidopsis*, mutation in a gynoecium-expressed phospholipase AII (*pPLAIIγ*) gene produces maternal haploid plants (Jang et al., 2023). Jiang et al. reported that the haploid plants carrying the maternal genome were triggered from the maternal parent rather than through the pollen. They further proposed that mutation in *pPLAIIγ* in the funiculus led to the altered localization of auxin transporter PIN, ultimately resulting in maternal haploid induction. This study did not rule out the possibility of the mutations in *pPLAIIγ* might also lead to ROS accumulation in the female reproductive structure, impairing pollen tube functions upon fertilization that could confer to the pollen a haploid inducing activity (Jang et al., 2023). The detailed molecular and genetic mechanisms underlying this HI strategy require further detailed studies, and it is also important to investigate whether the haploid seeds produced by this haploid inducer are the progeny of a single fertilization or double fertilization events.

**Table 1:** Maternal and paternal factors showing different haploid induction rates (HIR) in plant species.

Gene	Parental factor	Biological function	HIR	Species	Reference
*ZmPLA1/NLD/MTL*	Paternal	Phospholipase A	1-2%	Maize	Coe, 1959
	Paterna	Phospholipase A	6.70%	Maize	Gilles et al., 2017
	Paternal	Phospholipase A	5.86-15.7%	Wheat	Liu et al., 2020
	Paternal	Phospholipase A	6.34%	Rice	Jang et al., 2023
*ZmPOD65*	Paternal	Sperm-specific peroxidase	1-7.7%	Maize	Jiang et al., 2022
*ZmPLD3*	Paternal	Phospholipase D	0.85-0.96%	Maize	Li et al., 2021
*ZmDMP, ZMP8/9, CIDMP*	Paternal	Membrane localized protein	0.1-0.3%	Maize	Zhong et al., 2019
	Paterna	Membrane localized protein	1.03-4.4%	*B. napus*	Zhong et al., 2022b
	Paterna	Membrane localized protein	0.3-3.7%	Tomato	Zhong et al., 2022a
	Paternal	Membrane localized protein	1.1-2.1%	*Arabidopsis*	Zhong et al., 2020
	Paternal	Membrane localized protein	1.12%	Watermelon	Chen et al., 2023
	Paternal	Membrane localized protein	0.29-0.82%	*M. truncatula*	Wang N. et al., 2022
*KPL1/2*	Paternal	Ubiquitin E3 ligase	0.34%	*Arabidopsis*	Jacquier et al., 2023
*CENH3*	Paternal	Histone H3-like centromeric protein	34%	*Arabidopsis*	Ravi and Chan, 2010
	Paternal	Histone H3-like centromeric protein	14.29-50.38%	*Brassica crop*	Han et al., 2024
	Paternal	Histone H3-like centromeric protein	2.2-4.63%	Onion	Manape et al., 2024
	Maternal	Histone H3-like centromeric protein	2.04-2.68%	Onion	Manape et al., 2024
	Maternal	Histone H3-like centromeric protein	5%	*Arabidopsis*	Ravi and Chan, 2010
	Paternal	Histone H3-like centromeric protein	25%	*Arabidopsis*	Wang et al., 2023
	Maternal	Histone H3-like centromeric protein	77%	*Arabidopsis*	Wang et al., 2023
	Paternal	Histone H3-like centromeric protein	3.6%	Maize	Kelliher et al., 2016
	Maternal	Histone H3-like centromeric protein	3.6%	Maize	Kelliher et al., 2016
	Maternal	Histone H3-like centromeric protein	0.33-1%	Rice	Ahmadli et al., 2023
	Maternal	Histone H3-like centromeric protein	7%	Wheat	Lv et al., 2020
*pPLAIly*	Maternal	Phospholipase A II	1.07%	*Arabidopsis*	Jang et al., 2023
*ECS1/ECS2*	Maternal	Egg cell-specific aspartic endopeptidase	3.1-3.5%	Rice	Zhang et al., 2023
	Maternal	Egg cell-specific aspartic endopeptidase	0.8-1.1%	*Arabidopsis*	Zhang et al., 2023
*KNL2*	Maternal	Kinetochore proteins	10.5%	*Arabidopsis*	Ahmadli et al., 2023
*BBM*	Maternal	Transcription factors of AP2/ERF family	0.4-2.8%	*Arabidopsis*	Chen et al., 2022
	Maternal	Transcription factors of AP2/ERF family	0.4-3.55%	Maize	Qi et al., 2023
	Maternal	Transcription factors of AP2/ERF family	5-29%	Rice	Khanday et al., 2019
	Maternal	Transcription factors of AP2/ERF family	43.5-46.1%	Fern	Bui et al., 2017
	Maternal	Transcription factors of AP2/ERF family	1%	*B. napus*	Chen et al., 2022
*PAR*	Maternal	K2-2 zinc finger, EAR-domain protein	Not applicable	Lettuce	Underwood et al., 2022

A separate female *in vivo* HI strategy has been developed via the mutagenesis of two egg cell-specific aspartic endopeptidase (ECS) genes, *ECS1* and *ECS2* (Zhang et al., 2023). This HI system has been reported to work efficiently in both rice and *Arabidopsis* (**Table 1**). After sperm-egg cell fertilization, the two ECSs are required to ensure male and female nuclei fusion. Loss-of-function in *ECS1* and *ECS2* generates semigamous zygote (gamete fusion without nuclei fusion) with the sperm nucleus in the basal cell and the maternal genome in the apical cell which further divides to form a maternal haploid embryo. The haploids (0.13%) originated from the semigamous zygotes only took a small portion of the haploid pool (1%) of plants in the study, thus Zhang et al. speculate that chromosome elimination is an additional contributory mechanism. Of note, a similar conclusion from studying the same maternal factors but with 10.9% of HI rate was reported by another group which has also provided an alternative explanation to the underlying mechanisms (Mao et al., 2023). Previous studies have demonstrated that the endopeptidases ECS1 and ECS2, secreted by the fertilized egg cell, cleave the pollen tube attractant LURE to prevent polytubey (Yu et al., 2021). Moreover, ECS1 and ECS2 act as primary female factors that initiate the induction of maternal haploids. Double mutants of *ECS1* and *ECS2* exhibit delayed synergid disintegration, heightened susceptibility to hetero-fertilization, and give rise to haploid plants without a paternal genome contribution (Mao et al., 2023). Notably, the *ECS1* and *ECS2* mutants represent maternally inherited factors that induce the formation of maternal haploids, underscoring the significance of maternal effects in the phenomenon of haploid induction. However, further detailed evaluation and validations are required to fully understand the mechanism underlying the generation of haploid progeny from the *ecs1ecs2* double mutants.

The *BABY BOOM (BBM)* AINTEGUMENTA-LIKE (*AIL*) AP2/ERF domain transcription factor is a major regulator in early plant embryo and endosperm development. A gene encoding transcription factor Apospory-specific *Genome Region BabyBoom-like* (*ASGR-BBML*) *1*, was identified in parthenogenetic pearl millet (*Pennisetum squamulatum*) and has been reported to induce parthenogenesis in egg cells of sexual pearl millet (Conner et al., 2015). Further studies have shown that when *PsASGR-BBML1* is ectopically expressed in maize and rice egg cells, it can induce the formation of haploid embryos in both species (Conner et al., 2015, 2022; Qi et al., 2023). Interestingly, a rice ortholog of *PsASGR-BBML1*, known as *OsASGR-BBML1* (or *OsBBM1*), has also been found to stimulate division and development in rice egg cells without the need for fertilization (Khanday et al., 2019; Rahman et al., 2019). Apomixis is a unique form of asexual reproduction in flowering plants, resulting in seed formation without meiosis or egg fertilization. A key gene in this process, *PARTHENOGENESIS* (*PAR*), was discovered in parthenogenetic dandelions. This gene encodes a K2-2 zinc finger EAR-domain protein. When *PAR* is ectopically expressed in lettuce egg cells, it triggers the development of haploid embryo-like structures without fertilization (Underwood et al., 2022). This ability of *PAR* to induce embryo-like development was further demonstrated through its heterologous expression in lettuce egg cells. These findings provide valuable insights into the molecular mechanisms underlying plant reproductive processes and open new avenues for potential applications in crop breeding.

## 4 Epigenetically mismatched parental centromeres result in haploid offspring

Parental genome elimination causes haploid induction through the postzygotic loss of one of the parental chromosomes. A centromeric variant of histone 3 (*CENH3*)-based HI system where the modification of a *CENH3* gene allows the creation of HI lines was first demonstrated in *Arabidopsis* (Ravi and Chan, 2010). Mutations in *CENH3* can generate either maternal or paternal haploid seeds, respectively (Kuppu et al., 2015). Ever since, this *CENH3*-HI system has also been successfully applied in monocots, such as wheat and maize (Kelliher et al., 2019; Lv et al., 2020; Wang et al., 2021). It was suggested that the manipulation of CENH3 proteins results in slower chromosome movement during the first division of zygote, leading to the elimination of one set of parental chromosomes (Ravi and Chan, 2010). More detailed molecular and genetic mechanisms involved in this haploid production system have been documented in a later report in 2021 (Marimuthu et al., 2021). Alterations in the *CENH3* lead to its selective removal from the centromeres of mature egg cells and early zygotes, contrasting with the persistence of wild-type CENH3. Following double fertilization, hybrid zygotes exhibit a preference for loading CENH3 and essential centromere proteins onto the CENH3-enriched centromeres inherited from the wild-type parent. Conversely, centromeres depleted of CENH3 often fail to reconstitute new CENH3 chromatin and kinetochores, resulting in their frequent loss. This intricate process hints at the role of the E3 ubiquitin ligase VIM1 in opposing the genome elimination of *CENH3*-HI. Furthermore, the presence of parental *CENH3* polymorphisms contributes to the formation of epigenetically distinct centromeres, establishing a robust mating barrier that results in the production of haploids. These mechanisms underscore the complex interplay between *CENH3* alterations, chromatin dynamics, and reproductive developmental outcomes, shedding light on the intricate processes governing genome stability and inheritance in plants (Marimuthu et al., 2021). Additionally, a simple and highly efficient strategy was developed to induce both paternal and maternal haploids through ambient temperature manipulation in the *CENH3*-HI system (Wang et al., 2023). The enhancement of haploid induction at higher temperatures may be a common feature of *CENH3*-based haploid inducers. Kinetochore *Null2* (*KNL2*), an assembly factor of CENH3, can be used as a haploid inducer when mutated. Short-term temperature stress of the *knl2* mutant significantly increased the efficiency of haploid induction tenfold (Ahmadli et al., 2023), suggesting that haploid induction in centromere-impaired mutants is influenced by temperature stress during both ovule development and early embryogenesis.

## 5 Conclusions and perspective

Doubled haploid (DH) technology has emerged as a crucial tool in crop breeding, significantly improving efficiency across various plant species. This approach centers on generating haploid plants with a single parental genome, followed by chromosome doubling to produce genetically stable diploid plants. The resulting homozygous lines can be created much faster than through traditional breeding methods, which typically require multiple generations of crossing and selection. Upon obtaining haploid plants, the chromosome doubling process is initiated, effectively doubling the genetic material to yield genetically uniform diploid plants. This streamlined method has revolutionized plant breeding, offering researchers and breeders a powerful means to accelerate crop improvement programs (Ishii et al., 2016; Britt and Kuppu, 2016; Gilles et al., 2017; Tan et al., 2023).

Various methods can induce haploids, including gametophytic or androgenic induction, generation of plants from haploid tissues (*in situ* gynogenesis and androgenesis), and selective loss of a parental chromosome set via hybridization. The choice of method depends on the species, and haploid induction (HI) rates are influenced by factors such as gene interactions, parental factors, and environmental conditions (Ishii et al., 2016; Ahmadli et al., 2023; Wang et al., 2023). HI lines with varying haploid induction rates (**Table 1**), which are generally very low, are being continuously improved (Shen et al., 2015; Ahmadli et al., 2023; Wang et al., 2023; Zhang et al., 2023).

HI mechanisms generally involve parental genome elimination during early zygote division or egg cell parthenogenesis during seed development. The induction capacity of an inducer is gauged by the proportion of seeds with a haploid embryo out of the total number of seeds produced in cross-pollinations with a donor genotype. In maize, maternal haploid inducers exhibit significantly higher induction rates compared to paternal haploid inducers, making them the preferred choice for producing doubled haploid (DH) lines (Gilles et al., 2017). Overall, HI lines induce parental genome elimination during the initial cell division of the zygote or through egg cell parthenogenesis during seed development (Marimuthu et al., 2021; Sidhu et al., 2022). Deciphering the molecular and genetic roles of these HI factors holds promise for augmenting induction rates. Notably, haploid seeds resulting from HI crosses often contain a significant proportion of aborted seeds due to specific factors for example involved in endosperm development. Typically, normal embryo development requires coordination of proper endosperm development (Lafon-Placette and Köhler, 2014; Song et al., 2021b). A question is how the fertilization of central cell affect haploid seed development.

Meanwhile, HI lines serve as valuable resources for investigating gene functions in seed development. For instance, the *CENH3*-HI line has been used to study the role of endosperm-expressed *LEAFY COTYLEDON1* (*LEC1*) in seed development, allowing for the generation of unique genetic materials to elucidate tissue-specific gene functions (Song et al., 2021a). The study utilized the *CENH3*-HI line to explore the function of endosperm-expressed *LEAFY COTYLEDON1* (*LEC1*) in seed development. Following pollination of *CENH3*-HI with *lec1* mutant pollen, the maternal plant produced several haploid progenies with embryos (*lec1*-) containing a single copy of the genome from the *lec1* mutant, while the endosperm (*LEC1*++/-) inherited genetic information from both parents. This HI line enables the generation of unique genetic materials to elucidate the developmental roles of tissue-derived genes. Applications of plant genomics technologies have become a cornerstone in uncovering the relationships between phenotypes and genotypes. Nonetheless, initial diploid plants often display heterozygosity in altered regions, necessitating additional generations to achieve homozygosity. Proposed haploid strategies aim to expedite and reduce costs for validating plant gene functions (Shen et al., 2015). However, the broad adoption of haploid induction (HI) systems developed for one species applicable to other species is hindered by several biological or technical constraints (Shen et al., 2015).

Moving forward, a comprehensive understanding of reproductive and seed development programs could reveal more HI factors, potentially leading to the development of broadly applicable haploid induction systems. This knowledge will be crucial in advancing crop improvement strategies and accelerating the development of new, improved plant varieties.
